# Asymmetric Deep Medullary Veins in Patients With Occlusion of a Large Cerebral Artery: Association With Cortical Veins, Leptomeningeal Collaterals, and Prognosis

**DOI:** 10.3389/fneur.2019.01292

**Published:** 2019-12-05

**Authors:** Zhihua Xu, Yang Duan, Benqiang Yang, Xin Huang, Yusong Pei, Xiaoqiu Li

**Affiliations:** ^1^Department of Radiology, TongDe Hospital of Zhejiang Province, Hangzhou, China; ^2^Department of Radiology, Center for Neuroimaging, The General Hospital of Northern Theater Command, Shenyang, China; ^3^Department of Radiology, The General Hospital of Northern Theater Command, Shenyang, China; ^4^Department of Radiology, Shengjing Hospital of China Medical University, Shenyang, China; ^5^Department of Neurology, The General Hospital of Northern Theater Command, Shenyang, China

**Keywords:** susceptibility weighted imaging, cerebral veins, collateral circulation, deep medullary veins, cortical veins, prognosis

## Abstract

**Objective:** To explore the relationships of asymmetric deep medullary veins (ADMV) to asymmetric cortical veins (ACV), leptomeningeal collaterals and prognosis in patients with occlusion of a large cerebral artery.

**Methods:** Clinical and imaging data of 56 patients with occlusion of a large cerebral artery were collected and reviewed. We assessed the time delayed between stroke onset and MR imaging (within 24 h of stroke onset), extension of cerebral infarction using the Alberta stroke program early CT score based on diffusion-weighted imaging (ASPECTs). ADMV and ACV were assessed using susceptibility-weighted imaging. The presence of ADMV (ACV) was defined as deep medullary veins (cortical veins) of the affected hemisphere that were greater in number and diameter than in the contralateral hemisphere. To evaluate leptomeningeal collaterals, the hyperintense vessel sign (HVS) was detected using T2 weighted fluid attenuated inversion recovery images. At 90 days, a modified Rankin scale score (mRS) was assessed to evaluate the clinical outcome.

**Results:** Of 56 patients, 27 presented with ADMV. Those patients who presented with and without ADMV differed significantly in HVS and ACV (*P* < 0.05) but not in time delayed between stroke onset and MR imaging, age, gender, stroke risk factors, baseline NIHSS score, or modified Rankin scale score at 3 months (*P* > 0.05). Logistic regression analysis found that the presence of ADMV was independently related to HVS and ACV (ACV: OR 95% C.I., 1.287–4.368; HVS: OR 95% C.I., 1.132–4.887).

**Conclusions:** The presence of ADMV on SWI was associated with prominent ACV and good leptomeningeal collateral flow but was not related to prognosis in patients with occlusion of a large cerebral artery.

## Introduction

In recent years, due to the application of susceptibility weighted imaging (SWI) to patients who suffered from cerebral ischemic stroke, alterations of draining veins after ischemia or infarction are better understood and have attracted increasing research interest. Initially, asymmetric deep medullary veins (1, 2) (ADMV) and asymmetric cortical veins (3) (ACV) were found in SWI in cerebral stroke patients. Subsequently, some studies demonstrated that ADMV and ACV were correlated with lower cerebral blood flow or perfusion (1, 2, 4, 5), lower vascular reactivity (6), and even clinical outcome (7, 8).

Formerly, collateral arterial circulation was considered of great concern. Leptomeningeal collateral vessels are of interest, especially when a large cerebral artery is occluded. Leptomeningeal collateral flow is a very important and main mechanism of perfusion compensation and an independent risk factor for prognosis in patients with larger vessel occlusion (9, 10). Both draining veins and leptomeningeal collaterals were detected at the same time in some patients with cerebral infarction. However, it is unclear whether alterations of leptomeningeal arteries and draining veins were related in patients with cerebral ischemic stroke. Moreover, the role of leptomeningeal arteries and draining veins on clinical outcome has not been investigated thoroughly.

Occlusion of a large cerebral artery is an important cause of cerebral infarction. Cerebral infarction is a dynamic disease, the status of ADMV, ACV, and leptomeningeal collateral vessels may differ over the time delayed between the stroke onset and MR imaging. Thus, we aim to explore the relationship of ADMV to ACV, leptomeningeal collateral vessels and clinical outcome in patients with occlusion of a large cerebral taking the time delayed between the stroke onset and MR imaging into consideration.

## Materials and Methods

### Patients

**Figure 1:**
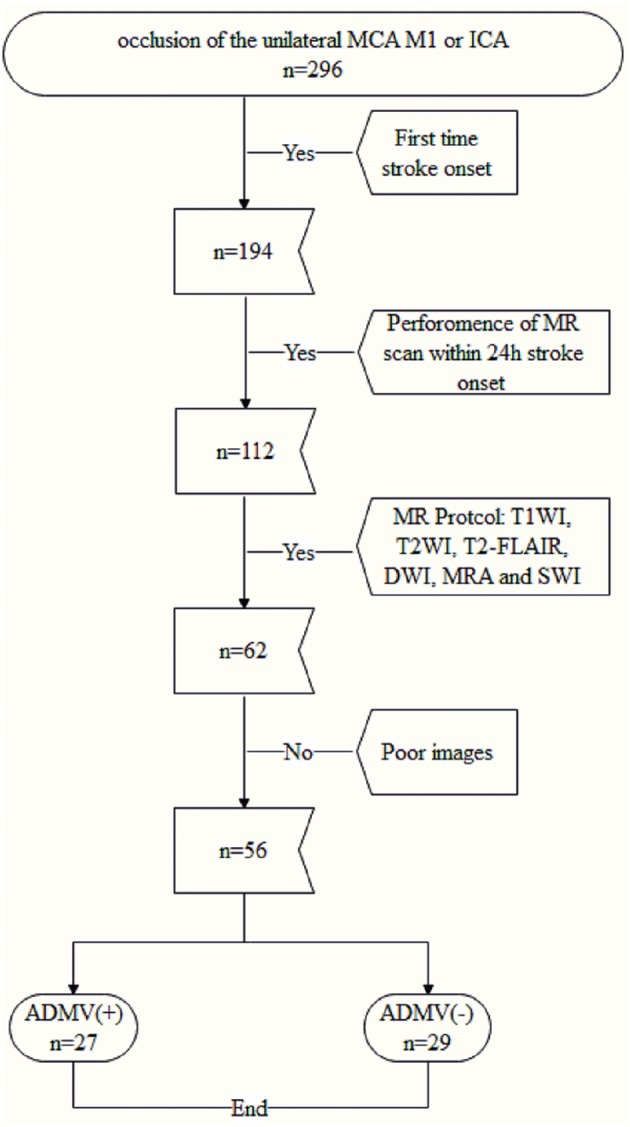
Flowchart of enrollment of study patients.

The protocol for this study was approved by the institutional review board of our hospital. All patients or their legally authorized representatives provided written informed consent prior to participation in this study. Fifty-six consecutive patients with occlusion of the unilateral proximal middle cerebral artery (MCA M1) or the internal carotid artery (ICA) were enrolled from January 2015 to October 2017 (Figure 1), and their clinical and image data were reviewed retrospectively.

The inclusion criteria were (a) occlusion of the unilateral MCA M1 or ICA confirmed by computed tomography angiography, vascular ultrasound or magnetic resonance angiography, (b) performance of magnetic resonance imaging including diffusion weighted imaging, SWI, and T2 weighted fluid attenuated inversion recovery (T2-FLAIR) sequences within 24 h after admission, and (c) first time stroke onset. The exclusion criteria were (a) abnormal brain MRI findings, such as space-occupying lesions, head trauma, hemorrhage, venous embolism, or infection and (b) poor image quality.

### Clinical Information

Stroke risk factors (hypertension, diabetes mellitus, hyperlipidemia, atrial fibrillation and current smoking) and the National Institute of Health stroke scale (NIHSS) of all patients were calculated on admission. The time delayed between stroke onset and MR imaging was recorded. At 90 days, the modified Rankin scale score (mRS) was assessed to evaluate the clinical outcome.

### MRI Protocol

All patients underwent multimodal MRI scanning, including a conventional sequence (T1WI, T2WI, and T2-FLAIR), diffusion-weighted imaging (DWI), MRA and SWI sequence on a 3.0T scanner (Discovery MR750; General Electric Healthcare, Chicago, IL, USA) using an eight-channel phased-array head coil. The relevant important parameters were (a) SWI: repetition time = 27 ms, echo time = 20 ms, flip angle = 10°, slice thickness = 2 mm, intersection gap = 0 mm, field of view = 24 × 24 cm2, and matrix number = 512 × 512 and (b) T2-FLAIR: repetition time = 8,800 ms, echo time = 94 ms, inversion time = 2,500 ms, slice thickness = 5 mm, intersection gap = 1 mm, field of view = 24 × 24 cm2, and matrix number = 512 × 512.

### Imaging Analysis

All images were reviewed separately by two neuroradiologists. Disagreements were resolved by consensus. First, we determined whether an acute cerebral infarction occurred due to the occlusion of a unilateral M1 MCA or an ICA. Then the extension of the acute cerebral infarction was assessed on DWI images based on a quantitative grading system, the Alberta stroke program early CT Score (11) (ASPECTs).

**Figure 2:**
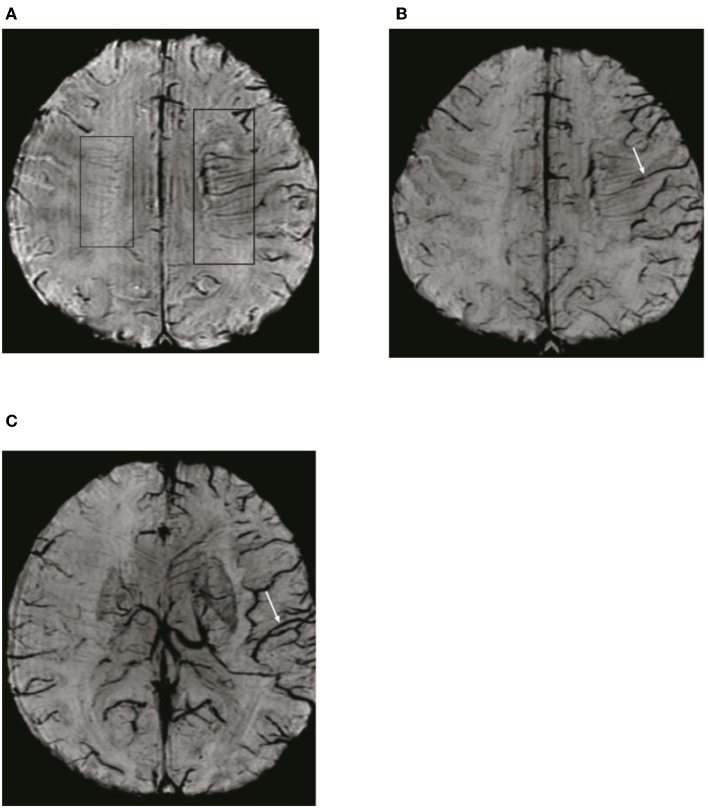
Illustration of asymmetric deep medullary veins (ADMV) and asymmetric cortical veins (ACV). **(A)** A greater number and diameter of deep medullary veins is seen in the left hemisphere than in the right hemisphere; this was defined as presence of ADMV. **(B,C)** Cortical veins of the left hemisphere were present in greater number and diameter in the regions of M1, M2, M3, I, M4, M5, and M6 than in the right hemisphere and was defined as ACV with a score of 7.

To detect ADMV and ACV, the SWI data were processed by minimal intensity projection with slice thickness of 10 mm. ADMV (ACV) was defined as deep medullary veins (cortical veins) of the affected hemisphere that were of greater number and diameter that in the contralateral hemisphere (8) (Figure 2). To quantify the ACV, the draining veins of the anterior circulation were divided into seven regions: I, insular ribbon; L, lentiform nucleus; M1, anterior middle cerebral artery (MCA) cortex; M2, MCA cortex lateral to the insular ribbon; M3, posterior MCA cortex; and M4, M5, and M6, anterior, lateral, and posterior, respectively, MCA territory immediately superior to M1, M2, and M3. Each region was scored 1 if ACV was present and 0 if ACV was absent on SWI. The total possible score of ACV was 7. A normal SWI had an ACV value of 0 points. A score of 7 indicated diffuse dilatation of cortical veins (Figure 2).

**Figure 3:**
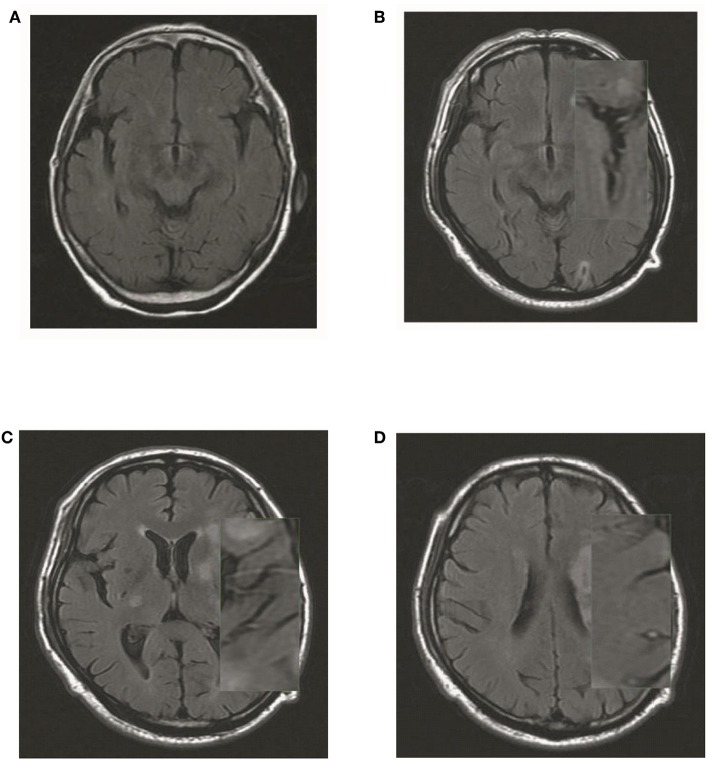
Illustration of HVS quantification. **(A)** A score of 0 was defined as absence of HVS; **(B)** a score of 1 indicated HVS limited to the Sylvian fissure; **(C)** a score of 2 indicated HVS limited to the Sylvian fissure and the cerebral sulci of the temporal-occipital junction; and **(D)** a score of 3 indicated HVS that extended to the cerebral sulci of the fronto-parietal lobe in addition the locations indicated by a HVS score of 1 or 2.

The hyperintense vessel sign (HVS) was assessed on T2-FLAIR images to reflect the status of leptomeningeal collateral vessels. A scoring system (9) ranging from 0 to 3 was used to quantify the HVS. A score of 0 was defined as absence of HVS; a score of 1 indicated HVS limited to the Sylvian fissure; a score of 2 indicated HVS limited to the Sylvian fissure and the cerebral sulci of the temporal-occipital junction; and a score of 3 indicated HVS that extended to the cerebral sulci of the fronto-parietal lobe in addition the locations indicated by a HVS score of 1 or 2 (Figure 3).

### Statistical Analysis

Categorical variables were described as frequencies and percentages. Continuous, normally distributed data were described as means and standard deviations (SD), and non-parametric data were described as medians and interquartile ranges (IQR). Interobserver agreement was assessed using the kappa statistic (κ); κ > 0.6 was considered good agreement, whereas κ > 0.8 was considered excellent (12). Differences in age, time delayed between stroke onset and MR imaging, NIHSS on admission, ASPECTs and ACV between who patients were observed with and without ADMV on SWI were analyzed using *t*-tests; their differences in HVS were assessed using a Mann-Whitney U test; and differences in location of larger vessel occlusion, gender, stroke risk factors, and clinical outcome were analyzed using Chi-square tests. Logistic regression analysis was performed to identify factors independently related to ADMV. Statistical significance was defined as *P* < 0.05. The data were analyzed using the Statistical Package for Social Sciences for Windows, Version 20 (IBM Corp., Armonk, NY, USA).

## Results

A total of 56 patients (36 males) with an MCA M1 or an ICA occlusion were enrolled in this study, and their mean age (SD) was 65.50 (9.39) years. At admission, their mean NIHSS (SD) and median GCS (IQR) were 8.66 (6.63) and 15 (12.5–15), respectively. According to MR images performed within 24 h after admission, their median ACV, HVS (IQR) and mean ASPECTs (SD) were 4 (2–5.7), 2 (0–2), and 5 (2), respectively. On discharge, their median mRS (IQR) was 3 (1–3).

### Time Delayed Between Stroke Onset and MR Imaging

The mean time delayed between stroke onset and MR imaging was 12.70 ± 5.15 h. When the patients performed the MR scan, the occlusion of MCA M1 or ICA was persistent confirmed by MRA. Of 56 patients, there was 35 cases with MCA M1 and 21 cases with ICA occlusion.

### Inter-reader Agreement in Evaluation of ADMV, HVS, and ACV

The MRI quality met the diagnostic standard and allowed the neuroradiologist to identify DMV, ACV, and HVS; images of some patients were excluded due to poor quality. The agreement between readers was excellent for ADMV and ACV on SWI (ADMV: κ = 0.901; ACV: 0.925) and for HVS on T2-FLAIR images (κ = 0.863).

### Factors Related to ADMV

**Figure 4:**
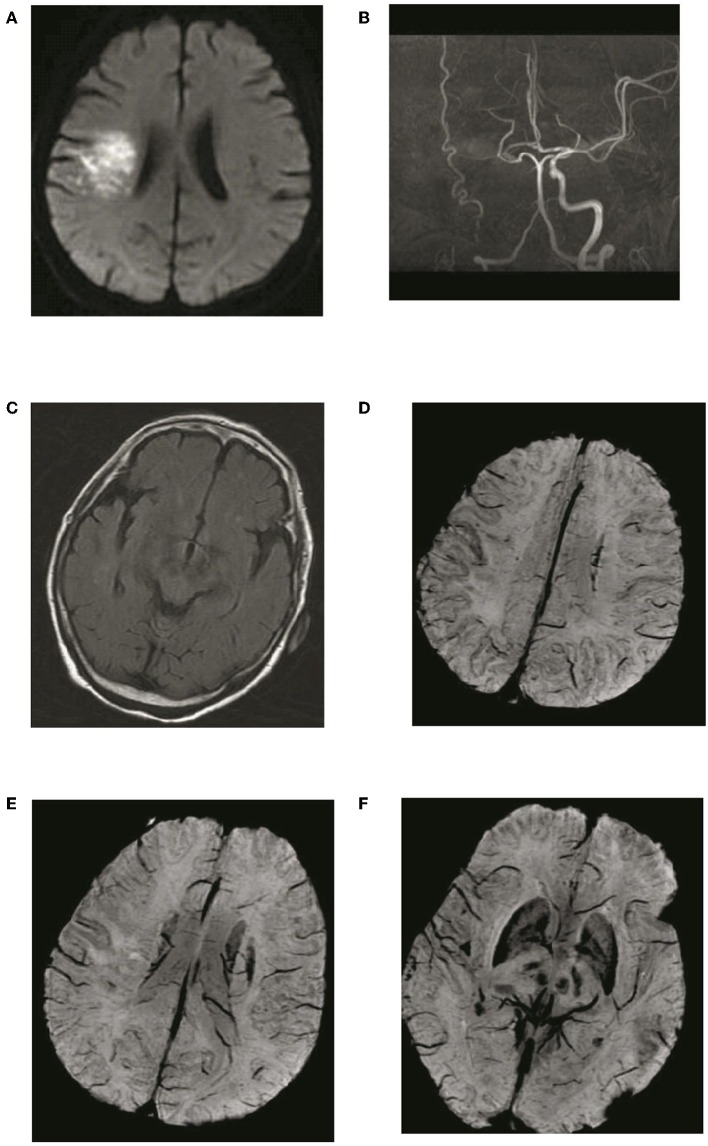
Typical patient with absence of asymmetric deep medullary veins (ADMV). A 69-year-old female presented with left limb weakness for 5 h. **(A)** The right parietal lobe shows acute corona radiata cerebral infarction on DWI; **(B)** Occlusion of the right internal carotid artery on magnetic resonance angiography with maximal intensity projection; **(C)** HVS with a score of 0 on T2-FLAIR image: **(D)** Absence of ADMV; **(E,F)** ACV with a score of 2 on SWI.

**Figure 5:**
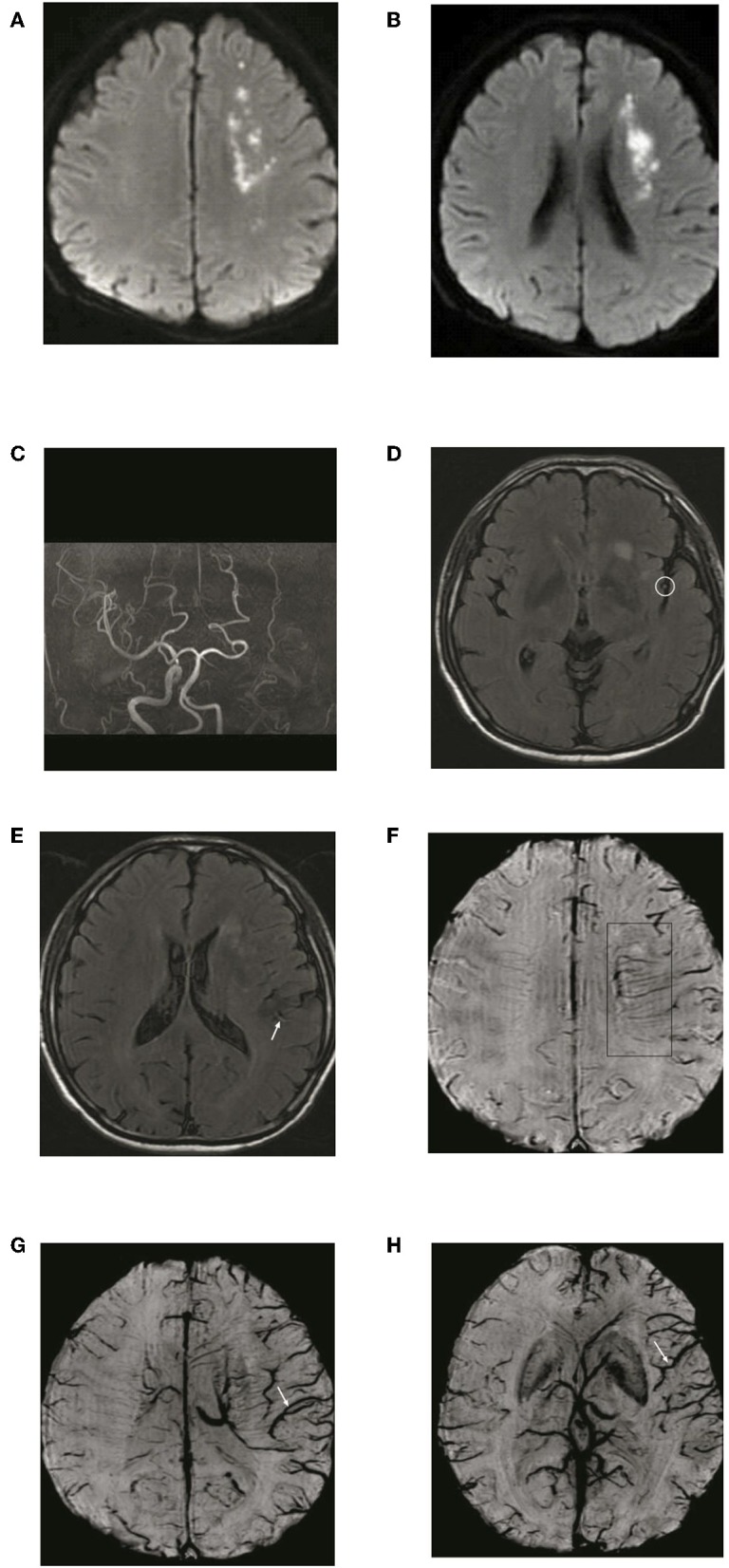
Typical patient with asymmetric deep medullary veins (ADMV). A 70-year-old male presented with right limb weakness for 6 h. **(A,B)** The left frontal lobe shows acute corona radiata cerebral infarction on DWI; **(C)** Occlusion of the left internal carotid artery on magnetic resonance angiography with maximal intensity projection; **(D,E)** HVS with a score of 2 on T2-FLAIR image: **(F)** Presence of ADMV; **(G,H)** ACV with a score of 6 on SWI.

Twenty-seven of 56 enrolled patient presented with ADMV. The relationships between ADMV and radiological and clinical features are shown in Table 1. ADMV were not associated with age, gender, stroke risk factors, the time delayed between stroke onset and MR imaging, location of larger vessel occlusion, ASPECTs, NIHSS on admission, or mRS at 3 months (*P* > 0.05). However, ADMV were associated with ACV and HVS (*P* < 0.05). In other words, those who presented with AMDV more frequently had higher ACV scores and HVS degree (Figures 4, 5) than those without AMDV.

**Table 1:** Univariate analysis of factors related to presence of ADMV in patients with occlusion of a large cerebral artery.

	**ADMV**		***P-*value**
	**Yes (*n* = 27)**	**No (*n* = 29)**	
Gender, M, *n*	19	17	0.359
Age, years	64.30 ± 9.03	66.62 ± 9.74	0.359
Stroke risk factors			
Hypertension, *n*	18	21	0.640
Hyperlipidemia, *n*	16	17	0.961
Diabetes mellitus, *n*	9	8	0.640
Current smoker, *n*	14	14	0.789
Atrial fibrillation, *n*	2	6	0.156
NIHSS	8.93 ± 6.19	8.41 ± 7.11	0.776
Radiological assessments			
HVS			0.015
0	4	15	
1	4	3	
2	14	7	
3	5	4	
ASPECTs	4.89 ± 2.10	5.28 ± 2.03	0.487
ACV score	5 (4–7)	3 (1.5–4)	0.001
Location			0.629
MCA M1	16	19	
ICA	11	10	
Time^*^, hours	11.85 ± 4.79	13.48 ± 5.12	0.239
mRS	3 (2–3)	2 (1–3)	0.215

* *The time delayed between stroke onset and MR imaging*.

After a logistic regression analysis, ACV and HVS were independently related to ADMV (ACV: OR 95% C.I., 1.287–4.368; HVS: OR 95% C.I., 1.132–4.887) (Table 2).

**Table 2:** Factors significantly related to presence of ADMV in patients with occlusion of a large cerebral artery based on logistic regression analysis.

	***P*-value**	**OR value**	**OR value (95% C.I.)**	
			**Lowest**	**Highest**
HVS	0.022	2.352	1.132	4.887
ACV	0.008	2.339	1.287	4.368

### ADMV and Prognosis

As shown in Table 1, the prognosis, based on mRS at 90 days, did not differ significantly between patients with and without ADMV based on SWI (*P* > 0.05).

## Discussion

In this study, we found that ADMV were independently related to ACV and HVS. However, ADMV were not associated with mRS at 90 days in patients with occlusion of a large cerebral artery. Thus, we hypothesized that ADMV could provide information about cortical veins and leptomeningeal collateral but did not predict prognosis in many patients with an occluded large cerebral artery.

Regarding ADMV and ACV, previous studies reached a consensus about their mechanism when the brain parenchyma is in ischemia. One mechanism is increased venous volume due to vasodilatation induced by regional ischemia or trench (13). The other mechanism involves uncoupling between the oxygen supply and demand in the hypoperfused tissue, resulting in a relative increase in deoxyhemoglobin and a relative decrease in oxyhemoglobin (14).

HVS on T2-FLAIR image indicates slow anterograde or retrograde leptomeningeal collaterals; the loss of flow-voids on FLAIR results from this sluggish blood flow (15). Many studies indicated that HVS is a non-invasive parameter indicative of both perfusion defects and leptomeningeal collaterals in patients with cerebral ischemic stroke (9, 16).

### ADMV and ACV, HVS

This study demonstrated that ADMV were frequently accompanied by prominent ACV in patients with occlusion of the MCA M1 or ICA. A possible explanations is that occlusion of the MCA M1 or ICA leads to a large area of brain parenchyma with hypoperfusion resulting in a large area of draining veins with a relatively increased ratio between deoxyhemoglobin and oxyhemoglobin.

This study also indicated that ADMV were related to HVS. In other words, the presence of ADMV on SWI was associated with good leptomeningeal collateral circulation. Therefore, we think that increased oxygen extraction fraction and good leptomeningeal collateral work together to compensate for ischemic brain parenchyma when a large cerebral artery is occluded.

Although ADMV, ACV, and HVS are mechanisms that compensate for ischemia, many studies have shown that they are also associated with decreased cerebral blood flow in the ischemic penumbra (17–19). Consequently, the more prominent ADMV or ACV, the larger is the area of the ischemic penumbra. Thus, the area of brain tissue with ADMV, ACV, and HVS is still in misery perfusion and is an at-risk brain tissue.

### ADMV and Prognosis

According to our results, we consider that ADMV could not predict clinical outcome in these patients with occlusion of the MCA M1 or ICA. Although this finding was consistent with the study of Payabvash et al. (20), the value of ADMV for prognosis is still controversial. Some other studies demonstrated that ADMV were related to poor clinical outcome (1, 7, 8, 21) in patients with occlusion of the MCA M1 or ICA. However, Baik et al. (22) reported a good clinical outcome [7 days NIHSS score (mean: 3, range: 0–7); 90 day mRS (mean: 0.2, range: 0–2)] for 19 patients suffering from a large cerebral artery occlusion with ADMV on SWI when successful reperfusion was performed.

**Figure 6:**
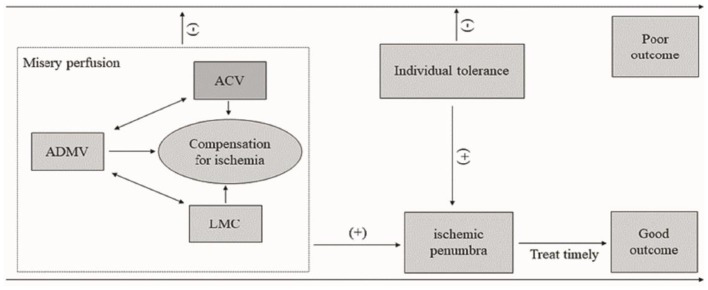
Diagram of the influence of ADMV, ACV, LMC, and ischemic tolerance on clinical outcome of acute occlusion of a large cerebral artery. An increased OEF (ADMV, ACV) and a good leptomeningeal collaterals (HVS) resulted in a misery perfusion. A misery perfusion and individual tolerance are positive for ischemic penumbra, and then a timely and successful recanalization to save the at-risk brain tissue may lead a good prognosis. Otherwise, the prognosis is poor. ADMV, asymmetric deep medullary veins; ACV, asymmetric cortical veins; LMC, leptomeningeal collateral; (−), negative or poor; (+), positive or good.

In our opinion, brain ischemia is a dynamic process. Collaterals and ischemia tolerances are the main independent risk factors for prognosis. Over time, the balance between collaterals, ischemia tolerances and cerebral parenchyma ischemic injury changes, which determines the prognosis. When collaterals and ischemia tolerances compensate for ischemic injury, the clinical outcome is good. Otherwise, the prognosis is poor (Figure 6).

Based on the aforementioned pathophysiological mechanisms of ADMV, ACV, and HVS, ADMV were associated with good collaterals, which can prolong the salvageable time of the penumbra. So, the good outcome experienced by some patients with ADMV is understandable. Parka et al. (23) reported a series of cases with an extensive ADMV and ACV, demonstrated by SWI, in ischemic brain tissues; however, diffusion-weighted imaging did not show any diffusion restriction lesion (named as “total mismatch of diffusion- and susceptibility-weighted imaging”) when a large cerebral artery was occluded. At 3 months, these patients had a good clinical outcome (mRS = 0). However, there is a chance that collaterals could collapse due to microvascular failure, and the integrity of good collaterals may not be maintained throughout the acute phase of cerebral ischemia. Study of ischemia in a rat middle cerebral artery occlusion model showed that rats still had reasonable functional recovery even when the brain was reperfused 14 days after occlusion (24).

Ischemia tolerance is an intrinsic neuroprotective mechanism whereby a brief, sublethal ischemic insult protects against a subsequent lethal ischemic attack (25). Although the precise mechanism by which ischemia tolerance affords protection to ischemic cerebral tissue remains undefined, the effects of ischemia tolerance on clinical prognosis have been demonstrated (26). Ischemia tolerance may be one of the explanations for why some patients with poor collateral have good clinical outcomes. Unfortunately, there is no tool to predict accurately ischemic tolerance now, which is worth future study.

Previously, studies of ADMV assessed at different time points after acute cerebral stroke resulted in different balances between collaterals, ischemia tolerances, and cerebral parenchyma ischemic injury and led to varied relationships to prognosis. Thus, we think that in our study ADMV could not predict clinical outcome for patients with occlusion of the MCA M1 or ICA but could reflect a status of misery perfusion, increased oxygen extraction fraction and good leptomeningeal collateral circulation. These patients did not always have poor clinical outcomes. Time is brain, a timely ischemic penumbra evaluation and successful recanalization would be essential to save the at-risk brain tissue.

Our study had some limitations. This study only enrolled a small sample of patients in a single center. Larger numbers of patients from multiple centers should be evaluated in the future. Infarct volume determined by an automated software or a manual delineation may provide a higher sensitivity and specificity than ASPECTS to assess the extension of cerebral infarction. Some patients were excluded for poor image quality and this may lead to bias. We did not perform digital subtraction angiography due high costs and invisibility and could not detect the status of leptomeningeal collaterals directly. Finally, dynamic observations of ADMV, ACV and HVS were not performed in the present study; they are worth performing in future studies.

## Conclusion

ADMV could reflect a status of misery perfusion, increased oxygen extraction fraction and good leptomeningeal collateral circulation. The presence of ADMV may not lead to a poor outcome for many patients with occlusion of a large cerebral artery.

## Data Availability Statement

All datasets generated for this study are included in the article/supplementary material.

## Ethics Statement

The studies involving human participants were reviewed and approved by Ethics Committee of General hospital of Northern Theater Command. The patients/participants provided their written informed consent to participate in this study.

## Author Contributions

ZX, YD, and BY conceived the project idea. XL provided critical suggestions for the experiments design and collected the clinical data and following-up. XH and YP collected the imaging data. ZX and YD provided the imaging analysis. ZX, YD, and XH wrote the paper. YD and BY supervised the project.

### Conflict of Interest

The authors declare that the research was conducted in the absence of any commercial or financial relationships that could be construed as a potential conflict of interest.
